# Impact of plantaris ligamentous tendon

**DOI:** 10.1038/s41598-021-84186-w

**Published:** 2021-02-25

**Authors:** Łukasz Olewnik, Piotr Karauda, Bartosz Gonera, Konrad Kurtys, R. Shane Tubbs, Friedrich Paulsen, Rafał Szymański, Michał Polguj

**Affiliations:** 1 Department of Normal and Clinical Anatomy, Chair of Anatomy and Histology, Medical University of Lodz, Lodz, Poland; 2 Department of Anatomical Dissection and Donation, Medical University of Lodz, Lodz, Poland; 3 Department of Neurosurgery, Tulane University School of Medicine, New Orleans, LA USA; 4 Department of Neurosurgery and Ochsner Neuroscience Institute, Ochsner Health System, New Orleans, LA USA; 5 Department of Anatomical Sciences, St. George’s University, West Indies, Grenada; 6 Institute of Functional and Clinical Anatomy, Friedrich Alexander University Erlangen-Nürnberg, Erlangen, Germany; 7 Department of Topographic Anatomy and Operative Surgery, Sechenov University, Moscow, Russia; 8 Department of Histology, Chair of Anatomy and Histology, Medical University of Lodz, Lodz, Poland

**Keywords:** Anatomy, Musculoskeletal system, Ligaments

## Abstract

There are countless morphological variations among the muscles, tendons, ligaments, arteries, veins and nerves of the human body, many of which remain undescribed. Anatomical structures are also subject to evolution, many disappearing and others continually emerging. The main goal of this pilot study was to describe a previously undetected anatomical structure, the *plantaris ligamentous tendon*, and to determine its frequency and histology. Twenty-two lower limbs from 11 adult cadavers (11 left, and 11 right) fixed in 10% formalin were examined. The mean age of the cadavers at death was 60.1 years (range 38–85). The group comprised six women and five men from a Central European population. All anatomical dissections of the leg and foot area accorded with the pre-established protocol. Among the 22 lower limbs, the PLT was present in 16 (72.7%) and absent in six (27.3%). It originated as a strong fan-shaped *ligamentous tendon* from the superior part of the plantaris muscle, the posterior surface of the femur and the lateral aspect of the knee joint capsule. It inserted to the ilio-tibial band. Histologically, a tendon and ligament were observed extending parallel to each other. A new anatomical structure has been found, for which the name plantaris ligamentous tendon is proposed. It occurs around the popliteal region between the plantaris muscle, the posterior surface of the femur, and the ilio-tibial band.

## Introduction

Increasing numbers of morphological variations have been observed within human body structures during recent years. While many are common, others are more difficult to identify, their occurrence being noted only in individual case reports. Two apposite examples are the classification of the five types of anterolateral ligament^1^, previously deemed a "thickening" of the joint capsule, and the determination of the frequency of the frenular ligament in the foot^2^. Several variants of the fifth quadriceps head of the thigh have also been discovered^3^: this begs the question of whether the quadriceps should be renamed as a *five-headed* muscle. Another very important recent discovery is the *Interstitium*. Careful examination of this new organ can help to elucidate the spread of cancerous lesions and even facilitate their treatment^4^. Such studies have yielded a wealth of information about morphological variations of existing structures and have even identified new ones. It is unclear whether this extensive morphological variety reflects changes during human evolution.

The popliteal region demonstrates extensive morphological variation in the muscular system^5–9^. The popliteal region and plantaris muscle are very variable and a tensor fasciae suralis can be present^5–7,9,10^. A comprehensive understanding of the morphological variations in this region is required for making correct diagnoses and planning effective treatment and rehabilitation. For example, any type of proximal attachment of the plantaris muscle can be associated with a range of conditions such as patellofemoral pain syndrome^9^. In turn, the type of tendon connection, or even the belly of the popliteal muscle itself, can influence the stability of the knee and its support by the popliteal ligaments or even the fibular collateral ligament^10,11^.

This work describes a previously-undescribed structure lying between the plantaris muscle, the popliteal oblique ligament and the ilio-tibial band, for which we propose the name plantaris ligamentous tendon (PLT). The name refers to both the ligamentous and tendinous features of the newly-found structure, which appears to always originate from the superior part of the plantaris muscle. Both tissue types were confirmed histologically. The aim of the present study was to examine the microscopic and macroscopic structure of the PLT and determine its incidence.

## Materials and methods

### Anatomical examination

Twenty-two lower limbs from 11 cadavers (11 left and 11 right) fixed in 10% formalin were examined. The mean age of the cadavers *at death* was 60.1 years (range 38–85), and the group comprised six women and five men from a Central European population. The cadavers were the property of the Department, following donation to the university anatomy program. Lower limbs with evidence of surgical intervention in the dissected area were excluded. All dissections of the leg and foot area accorded with the pre-established protocol^2,12,13^.

Dissection began with the removal of the skin and superficial fascia from the area of the knee and leg up to the gastrocnemius muscle. Following this, the lateral and medial heads of the proximal part of the gastrocnemius were separated from each other^13^. The medial head was then partially removed and the lateral head was separated at the muscular-tendon junction, thus exposing the proximal parts of the soleus and plantaris muscles. The next step was to clean the anatomical structures around the plantaris muscle^13^. Following this, the *plantaris ligamentous tendon* (PLT) was thoroughly cleaned and its attachments checked. In addition, an accessory band from the plantaris muscle was examined for any attachment to the iliotibial band, according to the classification proposed by Olewnik et al.^13^.

### Histological examination

After the PLT was collected, the slice was fixed in buffer (10% aqueous formalin) and then embedded in paraffin wax. The preparation was stained with hematoxylin and eosin to highlight the basic features of tissue architecture. The PLT and accessory band from the plantaris muscle sections were photographed using an Olympus CX43 light microscope and an Olympus EP50 camera. Pictures were taken at 25 × and 400 × magnifications. The histological examination was aimed at demonstrating the differences between the PLT and the accessory band from the plantaris muscle, which inserts to the iliotibial band.

### Preparation using the paraffin technique, and staining with hematoxylin and eosin

The material was collected and fixed in a 10% formalin solution. The tissues were then rinsed with distilled water, and then dehydrated using a series of alcohols with increasing concentrations from 50 to 100%. The dehydrated tissue was then washed three times in xylene and then soaked in liquid paraffin at 56 °C. After the paraffin blocks had cooled, they were cut into 5 µm-thick sections using a semi-automatic rotary microtome and applied to glass slides. The tissues were then washed three times with xylene and rehydrated with a series of alcohols of decreasing concentration (from 100 to 50%).

The slides with the sections were then rinsed with distilled water, and immersed in Meyer’s hematoxylin for 10 min. After the staining was completed, the tissues were rinsed for 15 min in running water. The sections were then stained in eosin solution for two minutes. After the staining was completed, the slides were rinsed in distilled water and dehydrated using a series of alcohols of increasing concentration (from 50 to 100%). Finally, the preparation was rinsed in xylene and sealed under a coverslip with a synthetic resin (DPX).

### Morphometric measurements

An electronic digital caliper was used for all measurements (Mitutoyo Corporation, Kawasaki-shi, Kanagawa, Japan). Each measurement was carried out twice with an accuracy of up to 0.1 mm.

### Statistical analysis

Nominal data were compared with the Chi^2^ test. Measurements of continuous variables were compared between body sides and sexes using the Wilcoxon test and the Mann–Whitney test, respectively.

All analyses were performed using the Statistica 13.1 software package (StatSoft, Cracow, Poland—Dell Inc. (2016). Dell Statistica (data analysis software system), version 13. software.dell.com.). A value of *p* < 0.05 was considered significant.

The methods and all were carried out with relevant guidelines and regulations strictly developed and provided by Medical University of Lodz. The protocol of the study was accepted by Bioethics Committee of Medical University of Lodz (resolution RNN/297/17/KE). The cadavers belong to the Medical University of Lodz.

### Ethical approval and consent to participate

The cadaver belonged to the Department of Anatomical Dissection and Donation, Medical University of Lodz. Cadavers come from the Informed Donation Program.

### Consent to publish

Not applicable.

## Results

### Frequency of occurrence of the PLT

The PLT was present in 16 of the 22 lower limbs (72.7%) and absent in six (27.3%). It was absent in one man and five women (*p* = 0.3118); two right limbs, and four left (*p* = 0.6576)—Table 1.

**Table 1:** A comparison of morphological parameters between body sides and sexes (mm).

Parameter	Body side		*P* value	Sex		*P* value
	Left	Right		Women	Men	
Distance from the joint line	24.2 (5.8)	18.8 (9.5)	0.3329	1.25 (0.42)	2.75 (0.27)	0.0017
PLT length	22.74 (1.76)	21.10 (2.82)	0.2725	19.28 (1.43)	23.69 (0.72)	0.0024
Width of the PLT at the attachment to the iliolumbar band	3.83 (0.43)	3.67 (0.46)	0.4777	3.30 (0.16)	4.07 (0.23)	0.0024
Thickness of the PLT at the attachment to the iliolumbar band	1.81 (0.25)	1.69 (0.26)	0.5613	1.48 (0.08)	1.95 (0.09)	0.0024
Width of the PLT at the attachment to the capsule and PM	6.93 (1.05)	6.52 (1.09)	0.4777	5.85 (0.45)	7.33 (0.92)	0.0118
Thickness of the PLT at the attachment to the capsule and PM	2.19 (0.35)	2.09 (0.34)	0.5613	1.82 (0.04)	2.37 (0.24)	0.0024

### Morphology of the PLT

**Figure 1:**
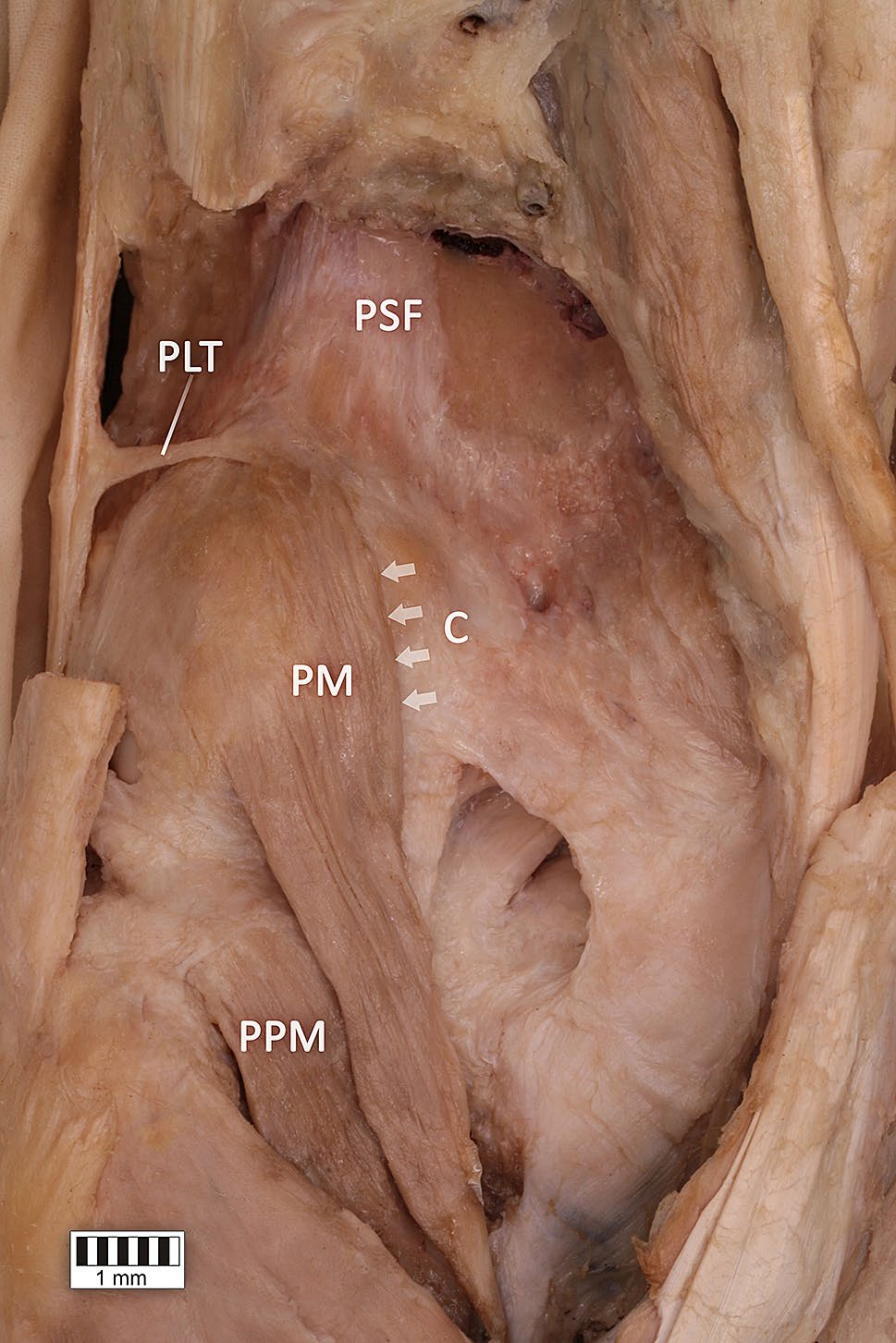
Plantaris ligamentous tendon. *PLT* plantaris ligamentous tendon; *PSF* posterior surface of the femur; *C* capsule of the knee joint; *PM* plantaris muscle; *PPM* popliteus muscle*. White arrowheads show the attachment of the plantaris muscle to the capsule of the knee joint.*

**Figure 2:**
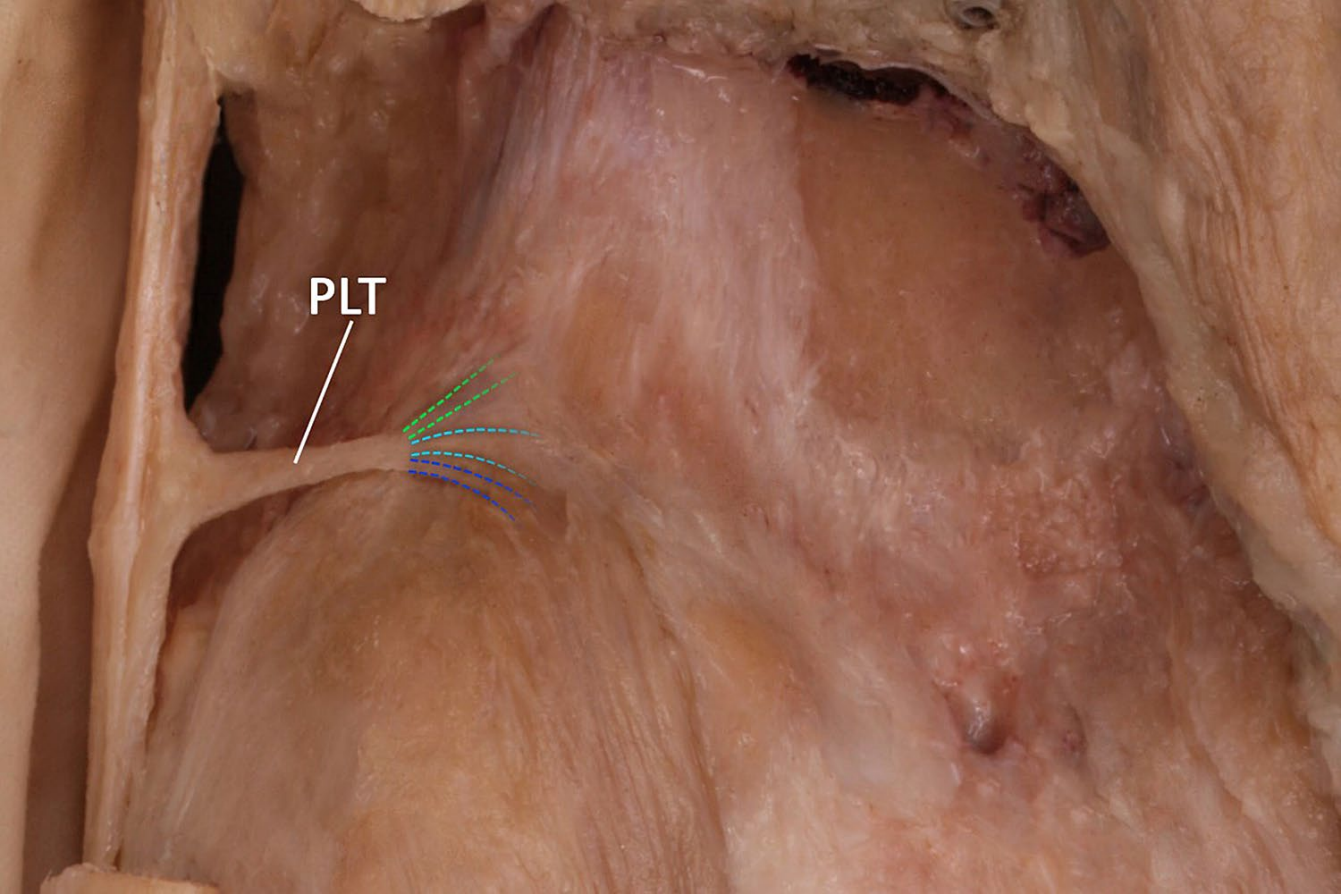
Proximal attachment of the plantaris ligamentous tendon. *PLT* plantaris ligamentous tendon. *Dotted green lines show attachment to posterior surface of the femur. Dotted turquoise lines show attachment to the capsule of the knee joint, while dotted blue lines show attachment to the plantaris muscle.*

The PLT originated as a strongly fan-shaped *ligamentous tendon* from the superior part of the plantaris muscle, the posterior surface of the femur and the lateral aspect of the capsule joint of the knee joint. It inserted to the ilio-tibial band (Figs. 1, 2). The site of attachment was 1–3 cm above the line of the knee joint.

### Histology of the PLT

**Figure 3:**
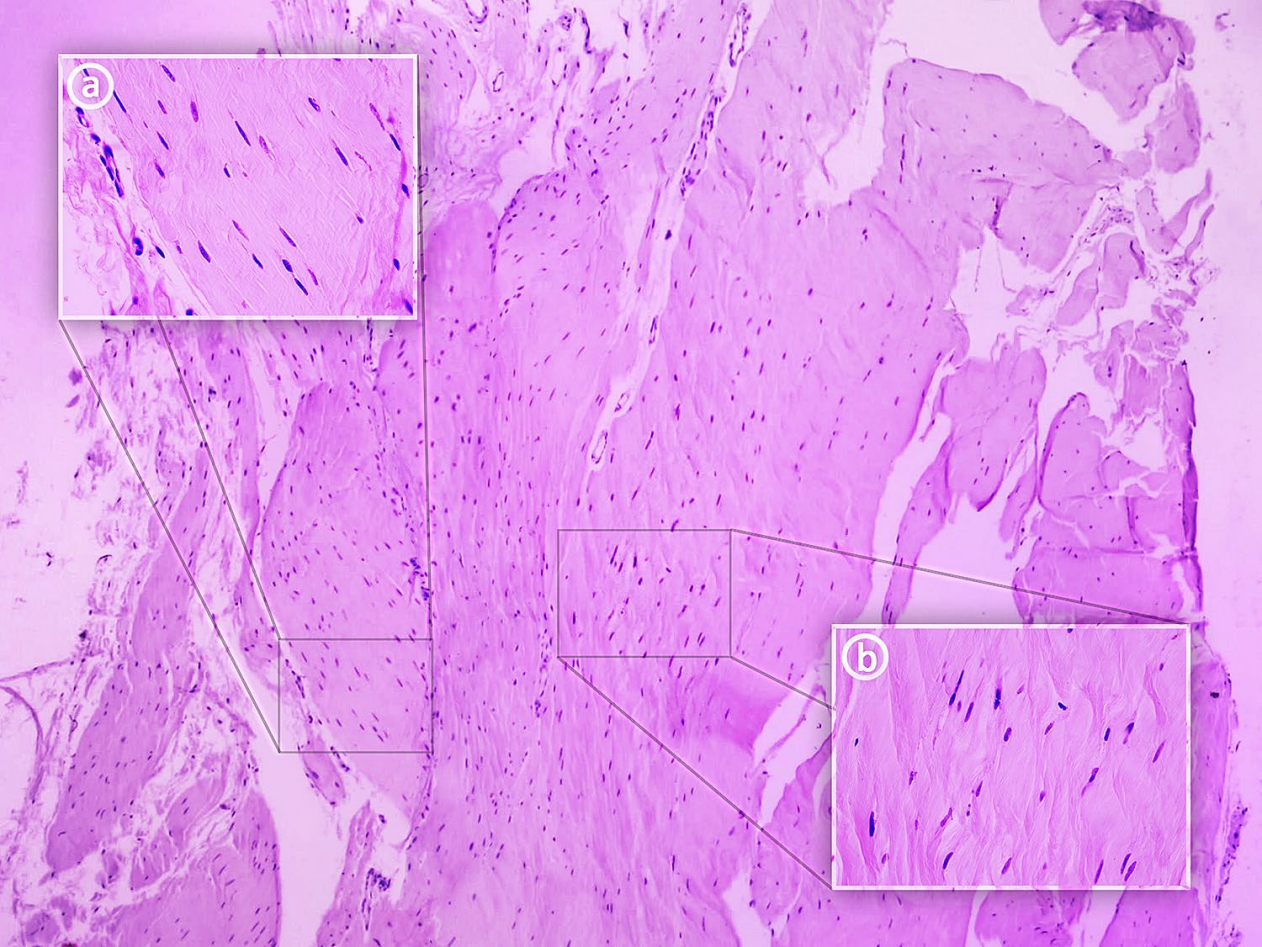
Histological examination of the plantaris ligamentous tendon. (**a**) 400 × magnification—tendon; (**b**) 400 × magnification—ligament.

Most connective tissue fibers are visible in one direction. Figure 3 shows the nuclei of connective tissue cells. A number of unstained spaces can be seen: these probably resulted from post-mortem tissue lysis and sample preparation. An image at 25 × magnification is presented together with close-ups at 400 × (a and b).

Figure 3a. 400 × magnification: An approximation of a fragment of the PLT can be seen, indicated by the course of the collagen fibers. The fibroblasts forming the parallel fibers contain elongated and flattened nuclei. An important feature of the architecture of the indicated tissue is that a small amount of basic substance can be seen between the collagen fibers. These qualities are all characteristic of tendons.

Figure 3b. 400 × magnification: The photograph shows an approximation of a fragment of the PLT. The collagen fiber system differs from that seen in Fig. 2, the fibers being arranged in multidirectional waves rather than lying close and parallel to each other. Fibroblast nuclei are visible in different planes between the collagen fibers. Their shape is mainly ovoid. This arrangement of collagen fibers and the nuclei shape are characteristic of the PLT ligament^14,15^.

### Frequency of accessory band of plantaris muscle and histological examination

**Figure 4:**
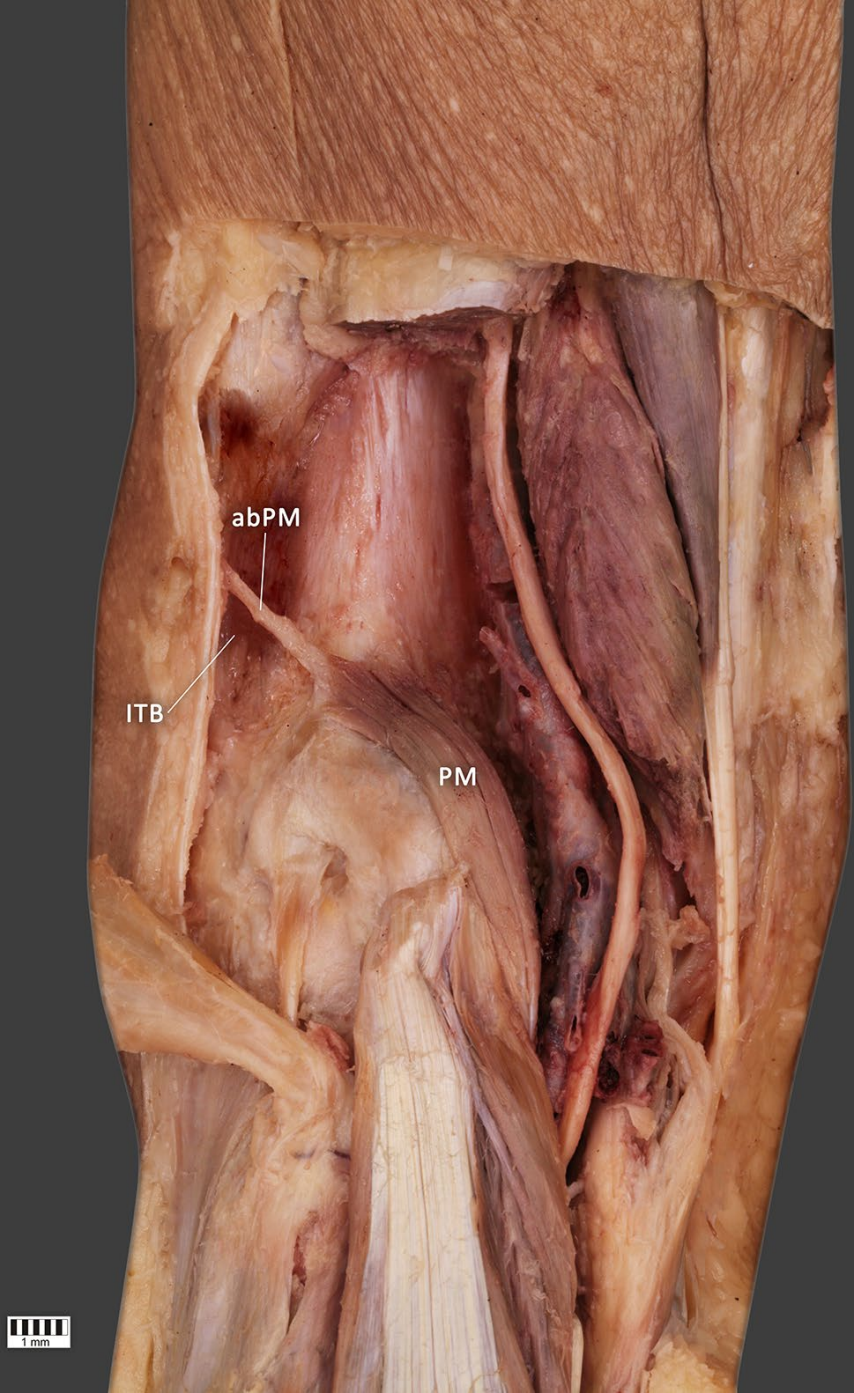
Type of origin of the plantaris muscle. (**a**,**b**) *PM* accessory band of the plantaris muscle. *PM* plantaris muscle; *ITB* iliotibial band.

The accessory band from the plantaris tendon, which inserts to the iliotibial band, was found in three lower limbs (13.6%)—Fig. 4. The PLT was absent from the lower limbs where there was an accessory band.

**Figure 5:**
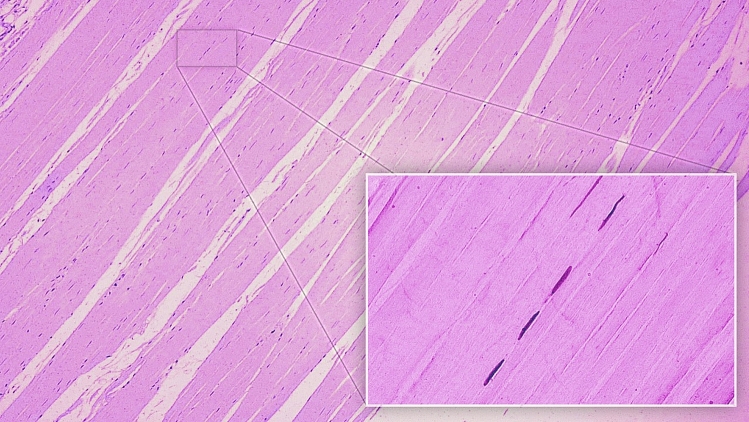
Histological examination of the accessory band of the plantaris muscle.

Most connective tissue fibers follow the same direction. Figure 5 shows the nuclei of the connective tissue cells. A number of unstained spaces can be seen: these probably resulted from post-mortem tissue lysis and sample preparation. An image at 25 × magnification is presented together with close-ups at 400 × (a and b). Histological examination revealed that the fibroblasts forming the parallel, closely-packed fiber bands contain flattened nuclei. An important feature of the architecture of this tissue is the presence of a small amount of basic substance visible between the collagen fibers; the shape of the fibroblast nuclei is also typical of tendons (Fig. 5).

## Discussion

This work presents the first anatomical and histological description of a new structure, for which we propose the name *Plantaris ligamentous tendon*. However, an important question remains: does it represent a residual/vestigial structure or a spontaneous variation?

The findings yielded by the recent growth in interest in the morphological variability of atavistic muscles in the lower limb, such as those concerning additional or absent muscles and new types of proximal and distal attachment, and variations in innervation and vascularization, indicate that human evolution is still continuing. Regarding the lower limb, the evidence suggests that the next stages of muscle evolution are being driven by the need to adopt a bipedal position. Because the force of gravity had to be focused on to only two limbs, the human femur developed an inward slope down to the knee, allowing the gluteal muscles to adapt to the stress and build the mass. This extra muscle allows balance to be maintained while standing on one leg and while “in-stride” during walking. The muscles near the ankle also help to provide a push during walking and running.

Originally, the plantaris muscle in the foot could have helped our distant ancestors to grab and manipulate objects as chimpanzees do now; however, with the passage of evolution, it has adapted to a new role by becoming so underdeveloped that it cannot be used to grip or grab anything. Concomitantly, the foot has become more elongated, and it is estimated that the plantaris muscle is now completely absent from 9 to 20% of humans^12,16^. Accordingly, the plantaris muscle is characterized by variability not only in incidence but also in insertion and origin. Our earlier work on the morphological variability of the proximal attachment of the plantaris yielded a six-fold classification. One subtype was characterized by the attachment of this muscle to the iliotibial band; in such cases, it could be confused with the PLT. In the present work, no concurrent PLT was observed, nor was there any band other than the iliotibial band from the plantaris muscle. The PLT differed histologically from the additional band of the plantaris muscle.

Many other such structures have been re-evaluated in recent years^1,11,17,18^. For example, studies suggest that the anterolateral ligament of the knee, rather than being a simple thickening of the joint capsule, could be an independent anatomical structure that occurs in several variants^1^. Similarly, the fifth head of the quadriceps femoris muscle was described as recently as 2016 as occurring in several variants^3^.

Additional muscles or slips from the medial or lateral head of the gastrocnemius, accessory soleus, fibularis quartus and fibularis digiti quinti have been noted much less frequently^19,20^. The tensor fascia suralis is one of the least frequently observed muscles in the lower limb^5–7^.

The PLT described herein was found in 16 of the 22 limbs (72.7%), indicating that it is a relatively “permanent” structure. Interestingly, it was more likely to be absent in women (5/11) than men (1/11). Despite this difference, it remains unclear whether the PLT is unique to humans, and indeed whether it has only formed recently as a response to our changing lifestyle. Further studies are needed to clarify this.

The PLT is a strong, fan-shaped “ligamentous tendon” which originates from the superior part of the plantaris muscle, the posterior surface of the femur and the lateral aspect of the capsule of the knee joint, and inserts to the iliotibial band. In addition, due to the relationship between its distal attachment and the iliotibial band, the PLT may possibly contribute to iliotibial band syndrome (ITBS). ITBS is a common injury caused by inflammation of the distal portion of the iliotibial band resulting in lateral knee pain. The distal iliotibial band slides over the lateral femoral epicondyle^9,13,21^. Excessive friction and potential irritation during repeated flexion and extension of the knee results in pain; it is therefore a frequent problem in the knees of athletes, especially endurance athletes, whose sport requires repetitive knee flexion^9,13,21^. Typical complaint of athletes with ITBS is a sharp or burning pain approximately 2 cm superiorly to the lateral knee joint line^9,13,21^. There is strong coincidence with our findings which identify the site of attachment of PLT at 1–3 cm above the knee joint. It, undoubtedly, suggests that this type of connection between PLT and iliotibial band may influence on the occurrence of ITBS. Additionally, fan-shaped attachments at the posterior surface of the femur, at the plantaris muscle and at the the knee joint capsule may possibly affect the tension of the joint capsule and interfere with plantaris muscle function.

Histological examination revealed a tendon and ligament extending parallel to each other. In Fig. 3a a small amount of basic substance can be observed between the collagen fibers. Furthermore, flattened and elongated nuclei are visible in the fibroblasts forming the parallel and closely-packed bands of collagen fibers constituting the PLT. These features imply that a tendon-like tissue is present in the PLT structure^14^. However, in Fig. 3b, the tissue is characterized by spheroidal fibroblasts and their mostly ovoid nuclei located between the fibers, which are arranged in multidirectional waves. This histological texture is more typical of ligaments^15^.

It is therefore necessary to consider whether this tissue represents a completely new histological structure or if the fibers could have become reorganised by posthumous changes in the examined tissue. Further immunohistochemical studies are needed to assess the exact protein composition of the basal connective tissue in the PLT, including glycosaminoglycans and the types of collagen in the fibers.

The present study has some limitations. The first is its lack of immunocytochemical examination. However, our research is a pilot study and is an introduction to a more thorough examination of the PLT. The second is the lack of sample size calculation; however, this is only the first study to describe PLT. The third limitation is the small research sample (22 limbs). Our findings could be useful in treating such conditions as ITBS, which could be caused by the PLT. Orthopedic surgeons and intervention radiologists, among others, should be very careful in this area.

## Conclusion

A new anatomical structure has been found within the popliteal region between the plantaris muscle, posterior surface of the femur and ilio-tibial band. It is proposed that it should be named the *Plantaris ligamentous tendon.* It is not a universal feature, being present in only 72.7% of the limbs studied. From a histological point of view, a tendon and ligament extending parallel to each other were observed. Without histological studies, the plantaris ligamentous tendon could be mistaken for an additional band of the plantaris muscle.

## Data Availability

Please contact authors for data requests (Łukasz Olewnik PhD—email address: lukasz.olewnik@umed.lodz.pl).
